# Risk Visualization With Petal-X and Continuous Glucose Monitoring to Support Person-Centered Care and Lifestyle Change in Cardiovascular Prevention: Qualitative Descriptive Study

**DOI:** 10.2196/92101

**Published:** 2026-10-13

**Authors:** Adrijana Svenšek, Diego Rojo, Mateja Lorber, Zalika Klemenc-Ketis, Lucija Gosak, Katrien Verbert, Gregor Štiglic

**Affiliations:** 1 Faculty of Health Sciences, University of Maribor, Žitna ulica 15, Maribor, 2000, Slovenia, 386 23004762; 2 Department of Computer Science, KU Leuven, Leuven, Flanders, Belgium; 3 Community Health Centre Ljubljana, Primary Healthcare Research and Development Institute, Ljubljana, Ljubljana, Slovenia; 4 Department of Family Medicine, Faculty of Medicine, University of Maribor, Maribor, Slovenia; 5 Department of Family Medicine, Faculty of Medicine, University of Ljubljana, Ljubljana, Slovenia; 6 Augment-IMEC Research Group at KU Leuven, Flanders, Belgium; 7 Susan Wakil School of Nursing and Midwifery, The University of Sydney, Sydney, New South Wales, Australia; 8 Faculty of Electrical Engineering and Computer Science, University of Maribor, Maribor, Slovenia; 9 Usher Institute, University of Edinburgh, Edinburgh, Scotland, United Kingdom

**Keywords:** cardiovascular disease, dashboard, continuous glucose monitoring, person-centered care, lifestyle change, qualitative content analysis, registered nurses

## Abstract

**Background:**

Cardiovascular diseases (CVDs) remain a leading cause of morbidity and mortality. In primary prevention, effective communication of individual risk and support for behavior change are crucial. Interactive risk visualizations can enhance understanding and engagement, while continuous glucose monitoring (CGM) offers immediate feedback on the physiological effects of daily choices.

**Objective:**

This study aimed to explore how patients at increased CVD risk and family medicine nurses perceive CVD risk visualization (Petal-X) and the integration of real-time glucose monitoring, with a focus on perceived person-centered care (PCC), motivation, and lifestyle change. This manuscript reports the qualitative phase of a larger sequential mixed methods study; the quantitative randomized controlled trial is reported separately.

**Methods:**

Using total population (census) sampling within a closed, predefined trial cohort, we invited every eligible patient who had completed the intervention arm of a parallel randomized controlled trial and every eligible registered nurse providing preventive consultations at the same single community health center in central Slovenia who had been familiarized with the intervention and with the Petal-X dashboard; 6 patients at increased CVD risk who had used the Petal-X dashboard in combination with CGM, and 6 nurses familiar with the intervention, took part, and interviews were conducted between September and December 2024. Data were collected through individual, face-to-face semistructured interviews that explored perceptions of the visualization, real-time glucose monitoring, and lifestyle change. Interviews were audio-recorded, transcribed verbatim, and analyzed by inductive qualitative content analysis within a qualitative descriptive design; coding continued until code saturation and data adequacy were judged to have been reached.

**Results:**

The analysis produced 11 subcategories, which were grouped into 4 categories, with an overarching theme running across them: visualization and digital tools as support for PCC to enhance motivation and lifestyle change in individuals at risk of CVD. Patients and nurses reported that personalized visual displays improved understanding of risk factors, facilitated dialogue and shared decision-making, and increased patients’ sense of involvement, responsibility, and control, although the 2 groups differed in emphasis: patients foregrounded personal meaning and self-regulation, whereas nurses foregrounded communication and fit with the consultation workflow. CGM was perceived as particularly motivating by linking behavior (food, activity, and stress) to real-time glucose changes, encouraging dietary and activity adjustments that some participants maintained after the intervention. Key barriers included variable digital literacy (especially among older users), fear or mistrust of technology, the attentional burden of continuous monitoring, potential information overload, workflow integration needs, and affordability or accessibility of CGM.

**Conclusions:**

Interactive CVD risk visualization Petal-X, especially when complemented by CGM, was perceived as a meaningful support for PCC and a driver of motivation and lifestyle change in primary prevention. Implementation should address training, usability, integration into routine workflows, and equity of access.

## Introduction

Primary health care in Slovenia is based on a network of 63 community health centers organized into 10 administrative regions, with approximately 900 teams of which around 200 operate privately under concession [1]. Family medicine practices offer both preventive and curative services. The preventive services are focused on individuals aged 30 years and older with no chronic disease, where registered nurses (RNs) assess the risk of developing chronic conditions [2,3]. In this process, prognostic models are used, which combine multiple predictive factors into a mathematical equation to estimate the likelihood of an outcome [4,5]. One of these is the SCORE2 (Systematic Coronary Risk Evaluation 2) model for estimation of cardiovascular disease (CVD), validated on the European population, which includes age, sex, smoking status, systolic blood pressure, total cholesterol, and high-density lipoprotein cholesterol [6]. Such models enable the early identification of high-risk individuals and guide the allocation of limited health care resources where they can achieve the most significant impact [7,8].

Nevertheless, CVDs remain the leading cause of death worldwide, accounting for over 20 million deaths each year [9]. Many individuals do not recognize how their daily habits (diet, physical activity, and smoking) directly influence their long-term CVD risk [10]. Therefore, effective communication of risk and the potential benefits of lifestyle change play a critical role in CVD prevention [11].

Visual tools are becoming an increasingly important approach in health care communication, as graphical and interactive representations of data improve understanding and foster motivation for lifestyle modification [9]. Digital health solutions that present personalized risk and the potential effects of change can help patients perceive their health status more tangibly. The growing digitalization of health care offers new possibilities for integrating real-time data with personalized risk assessment [12,13]. Continuous glucose monitoring (CGM) devices, for example, provide patients with immediate feedback on how their dietary and behavioral choices affect their blood glucose levels [14,15]. When combined with visualizations of long-term CVD risk, such as those provided by digital tools, this integration can support shared decision-making, enhance patient engagement, and promote more timely and sustainable lifestyle changes [16,17].

In this context, we examined the Petal-X visualization tool, which is based on the Petal Product Plots visualization and an explanation layer on top of the SCORE2 model. Its accuracy is comparable with the graphical score charts used in clinical practice. The developers of Petal-X conducted a study with nursing students, finding that its use improved understanding of risk factors and stimulated reflection on possible lifestyle changes [18].

The aim of our qualitative study was to explore how visualization of CVD risk, combined with the possibility of real-time blood glucose monitoring, influences the perception of person-centered care (PCC) among patients at increased risk of CVD. Furthermore, we sought to understand how RNs and patients perceive the use of these digital tools, as well as the opportunities and challenges they identify for their integration into everyday clinical practice.

## Methods

### Research Design

This research forms the qualitative phase of a sequential mixed methods design [19], carried out after the quantitative phase. The results of the quantitative analysis reported by Svenšek et al [20] indicated certain patterns that we wanted to explore in more detail. For this phase, we used a qualitative descriptive design [21], with data analyzed by inductive qualitative content analysis [22,23]. This approach was chosen because the aim of the study was descriptive and evaluative rather than theory-generating: we set out to characterize how patients who had completed the intervention arm, and the nurses working in the same preventive service who had been familiarized with it, experienced the Petal-X dashboard and CGM, including perceived usability, the perceived contribution to PCC and to motivation, and the barriers to implementation in routine practice. The study was not designed to develop a theory of a social or clinical process, and no theory-generating claims are made. We collected the data through individual semistructured interviews. We ensured the rigor of the qualitative study by adhering to Lincoln and Guba’s [24] criteria of credibility, dependability, confirmability, and transferability. Procedures are reported in accordance with the COREQ (Consolidated Criteria for Reporting Qualitative Research) 32-item checklist [25].

### Setting, Participants, and Recruitment

The study was conducted at a single community health center in central Slovenia that delivers preventive family medicine services. It was nested within a parallel randomized controlled trial (ClinicalTrials.gov NCT06226948) evaluating interactive digital visualization counseling for patients at risk of CVD [20]. Recruitment for the interviews was open to individuals across Slovenia, although the promotional campaign and study implementation were concentrated at this center. Patients were drawn from the trial intervention arm (n=30 at baseline), in which participants had a mean age of 51.9 (SD 7.2) years, 70% (21/30) were women, and the mean baseline 10-year SCORE2 risk was 4.87 (SD 2.61) and the highest of the 4 trial arms [20].

Patients were eligible if they were aged 40 years or older, had no diagnosed CVDs, and had at least 1 CVD risk factor (elevated systolic blood pressure, elevated fasting glucose, elevated blood lipids, or elevated BMI); nurses were eligible if they provided preventive consultations at the participating center, had been familiarized with the Petal-X dashboard and with CGM-based monitoring during the study period, and had at least 5 years of professional experience as a nurse, which was verified individually for each nurse before inclusion. Eligible patients who had completed the trial intervention arm were identified from the trial records by the study team, and eligible nurses were identified through the participating center; potential participants were then contacted individually by the first author (AS), in person or by telephone, informed about the aim and the voluntary nature of the study, and, if they agreed, gave written informed consent before an interview was scheduled. Recruitment was not purposive. We used a total population (census) approach within the closed, predefined cohort created by the parent trial: every patient who had completed the trial intervention arm and every nurse at the participating center who had been familiarized with the intervention was invited, and no further selection step was applied within this eligible pool, either on the basis of participant characteristics (eg, age, sex, and baseline risk category) or on the basis of the content of their experience. The size and composition of the sample were therefore determined by the boundaries of the parent trial and by willingness to participate, rather than by deliberate selection for particular characteristics, by maximum variation, or by an emergent analytic rationale. No more than 2 interviews were conducted per week so that each interview could be analyzed before the next. The final sample comprised 6 nurses and 6 patients.

### Definition of Intervention Completion and Extent of Exposure

Because eligibility for interview depended on having completed the intervention arm, we define here what completion involved in this study, independently of the report of the parent trial [20]. The intervention arm did not consist of a single encounter with the dashboard. Each patient attended a face-to-face preventive consultation, delivered by the first author (AS), an RN and PhD candidate, during which the patient’s individual 10-year SCORE2 risk was calculated and explored interactively using the Petal-X dashboard (duration of consultation: 30 minutes). Each patient was then fitted with a CGM sensor, which was worn during everyday life for 10 days, with real-time access to their own glucose readings through the associated smartphone app, and attended a further study visit 6 months later, at which the glucose record and the risk profile were reviewed together. The intervention therefore comprised both a nurse-led visualization consultation and a defined period of CGM-based self-monitoring. Patients were counted as completers of the intervention arm and were therefore eligible to be approached for an interview if they attended all 3 scheduled study visits (the baseline assessment, blood sampling at 3 months, and the final assessment at 6 months), wore the CGM sensor for at least 5 of the 10 intended days, and completed the final study assessment. Patients who withdrew, who discontinued sensor wear before this threshold, or who did not attend the final assessment were not counted as completers and were not invited to interview. Of the 30 patients randomized to intervention group 1 (Petal-X visualization combined with CGM), 27 met this definition of completion and were invited, and 6 agreed to be interviewed. Interviews were conducted a median of 5 weeks after the end of each patient’s participation, so that all patients described a completed rather than an ongoing experience of the intervention.

For nurses, participation did not involve delivering the intervention. All trial consultations, including the presentation of the Petal-X visualization and the application and explanation of the CGM sensor, were carried out by the first author (AS), and the participating nurses had no formal delivery role in the trial. They were RNs providing routine preventive consultations at the same center who had been familiarized with the intervention during the study period: each nurse took part in a single structured familiarization session of approximately 30 minutes, in which the Petal-X dashboard was presented and explored with example risk profiles, the rationale of the SCORE2-based visualization was explained, and the principles and practical use of CGM-based monitoring were demonstrated. Within this session, the nurses also used the dashboard themselves on example profiles, so that their appraisals were based on direct, if brief, hands-on contact with the tool rather than on observation alone. They also worked alongside the trial in the same preventive service and were therefore familiar with how it was organized and with the patients taking part. At the time of the interview, each nurse had approximately 7 months of familiarity with the tool and the intervention, which was delivered between May and December 2024, and the dashboard was displayed again at the start of the interview so that the discussion could refer to specific features of the visualization.

In this study, therefore, “completion” has a different meaning for the 2 groups. For patients, it denotes completion of the intervention arm as defined in the section Setting, Participants, and Recruitment; for nurses, it denotes completion of this familiarization and continuous presence in the setting for the whole intervention period, rather than a threshold of sessions delivered. Their accounts should accordingly be read as the informed appraisal of experienced preventive care nurses who knew the intervention and the tool well, and who work daily with the paper-based SCORE2 charts that the dashboard is intended to replace, rather than as the reflections of practitioners who had themselves delivered it in routine care; this distinction is revisited in the Limitations section.

### Data Collection

Interviews were conducted by the first author (AS), a female RN holding a master’s degree (MSc), who was trained in qualitative research methods and was a doctoral researcher at the University of Maribor and teaching assistant at the time of the study. The interviewer had no prior clinical or supervisory relationship with the participants before the study commenced. Participants were informed of the researcher’s professional and academic background and that the interviews formed part of a doctoral research project on digital tools for cardiovascular risk communication and prevention. The researcher’s background in nursing and digital health shaped an interest in PCC and technology-supported prevention; to limit the influence of these assumptions, reflexive memos were kept, and the developing interpretation was discussed in regular peer-debriefing meetings within the research team.

We conducted interviews with RNs who had been familiarized with the Petal-X dashboard for CVD risk visualization and with the potential for real-time blood glucose monitoring during the study period; at the start of each interview, the dashboard was displayed again so that the discussion could refer to specific features of the visualization. The patients we interviewed had all taken part in the intervention arm that combined the Petal-X dashboard with real-time monitoring of blood glucose levels. We were interested in the participants’ perceptions of the intervention and the use of new technologies. We used semistructured interviews to preserve certain concepts [24]. With the help of semistructured interviews, we obtained all the necessary information for the research, allowing participants as much freedom as possible in their responses. We opted for semistructured interviews because we did not want to stray from our topic and wanted to obtain an accurate description and perception of the participants’ involvement in interventions and visualizations related to CVD risk. With semistructured interviews and semi–open-ended questions, we also aimed to gather information about the experiences, feelings, and advice of RNs and research participants regarding the visualization of the risk of CVD using Petal-X and the potential for real-time monitoring of blood glucose levels. The interview guides for nurses and for patients are provided in Multimedia Appendices 1 and 2.

### Data Analysis

All interviews were audio-recorded, transcribed verbatim, and anonymized prior to analysis. Interviews were conducted and transcribed in Slovenian, and all coding and analysis were carried out in Slovenian to stay close to participants’ original wording; the illustrative quotations presented in the Results section were subsequently translated into English by the bilingual research team and checked against the original transcripts for accuracy and meaning. Data were analyzed by inductive qualitative content analysis [22,23]. Analysis proceeded alongside data collection, so that preliminary observations could inform the emphasis given to particular questions in later interviews.

Each transcript was first read repeatedly to obtain a sense of the whole. Meaning units relevant to the aim of the study were then identified and condensed, and each condensed meaning unit was labeled with a code that stayed close to participants’ own wording. The first transcripts were coded independently by 2 researchers (AS and LG), who compared their code lists and agreed on a shared coding frame; this frame was applied to the remaining transcripts and revised whenever new codes were added. Codes were compared systematically within and across the 2 participant groups and sorted, on the basis of similarity in content, into subcategories and then into categories. Finally, an overarching theme expressing the thread of meaning that ran across the categories was formulated. The developing category structure was reviewed and refined together with a senior researcher (GS) in peer-debriefing meetings until consensus was reached. Categories were refined iteratively by writing analytic memos, revisiting transcripts, and actively searching for discrepant accounts (eg, accounts of CGM worry or of limited benefit for some users). An audit trail (coding memos and versioned code lists) was maintained. To strengthen credibility, verbatim transcripts were returned to participants for review and possible correction (member checking).

Because the sample was bounded by the closed intervention arm of the parent trial, the size of the sample was fixed in advance rather than determined by the progress of the analysis. We therefore considered code saturation and the adequacy of the data for the descriptive aim of the study [26]. Toward the end of data collection, later interviews were adding few, if any, new codes, and the research team judged the material sufficient to describe the categories reported. We present this as an assessment of data adequacy rather than as a formal claim of saturation, since the number of available participants was fixed by the parent trial. In the Results section, quotes are attributed using participant identifiers to preserve anonymity: P denotes patients and N denotes RNs (eg, P1, N2). Each quotation is additionally accompanied by brief, nonidentifying contextual information about the speaker, namely, sex, age, and baseline 10-year SCORE2 risk category for patients, and years of professional experience for nurses, so that readers can interpret each quotation in relation to the individual speaking rather than only in relation to the participant group.

### Qualitative Rigor

Qualitative rigor was addressed using established criteria for qualitative rigor: credibility, dependability, confirmability, and transferability. Credibility was supported by verbatim transcription, systematic comparison of codes within and across the 2 participant groups, member checking (returning transcripts to participants for review and possible correction), regular peer-debriefing meetings within the research team, and the deliberate search for and analysis of negative cases. Dependability and confirmability were supported by maintaining an audit trail of coding memos and versioned code lists, so that the development of the codes, subcategories, and categories could be traced back to the underlying data. Transferability was supported by providing a thick description of the setting, participants, and context, enabling readers to judge the applicability of the findings to other primary care settings.

### Ethical Considerations

The study was approved by the Ethics Committee for Nursing Research at the Faculty of Health Sciences, University of Maribor (approval number 02/7K-2023), and the National Medical Ethics Committee of the Republic of Slovenia (approval number 0120-217/2023/4). We have also registered the research protocol of the clinical trial (ClinicalTrials.gov ID: NCT06226948) [27]. All participants were informed that their data would be used exclusively for research purposes. All participants received written and oral information about the study and gave written informed consent before their interview, including explicit consent to audio-recording and to the use of anonymized verbatim quotations in publications. Recordings were transcribed verbatim and deidentified; direct identifiers were removed and participants are referred to only by coded identifiers (P for patients and N for nurses). Data were stored securely, were accessible only to the research team, and were used exclusively for research purposes. Participants did not receive any financial compensation for taking part in the study.

## Results

### Participant Characteristics

The sample comprised 6 RNs and 6 patients at increased CVD risk, all from a single community health center in central Slovenia. All patients had taken part in the intervention arm of the parallel randomized controlled trial [20] that combined the Petal-X dashboard with CGM; they were aged 40 years or older, had no diagnosed CVD, and had at least 1 CVD risk factor. Among the 6 patients interviewed, 4 were women and 2 were men, aged 46‐67 (mean 57.8, SD 7.8) years; at baseline, 1 was at low, 2 at moderate, and 3 at high 10-year SCORE2 risk. The interviewed patients were therefore somewhat older than the intervention arm as a whole, in which the mean age was 51.9 (SD 7.2) years [20]. All 6 participating nurses were women, aged 26‐50 (mean 33.3, SD 10.2) years, and each had at least 5 years of professional experience as a nurse and worked in preventive family medicine consultations at the participating center throughout the trial period. To avoid duplication, participant characteristics are reported in the text only; brief individual descriptors accompany each quotation so that quotations can be read in relation to the individual speaker.

The analysis produced 11 subcategories, which were grouped into 4 categories (Table 1). Running across all 4 categories was an overarching theme, formulated as “Visualization and digital tools as support for PCC to enhance motivation and lifestyle change in individuals at risk of CVD.”

**Table 1.** Display categories and subcategories with codes.

Categories and subcategories	Codes
Person-centered care	
Goal-oriented care and individual approach	Time devoted to the patient; patient-centered care; tools as a means of improving treatment; individualized approach, and treatment; daily routine; meal planning; recommendations for the use of technology; involvement of family members; faster task completion; and ease of treatment.
Involvement in treatment	Patient involvement, treatment planning, increased sense of responsibility, sense of control, active role of the patient, and quality of treatment.
Knowledge and monitoring of risk factors for the development of CVD^a^	
Monitoring of vital functions and detection of changes or deviations	Real-time monitoring of blood glucose levels, monitoring of risk factors, changes in monitored vital signs, immediate monitoring of condition, detection of changes, and faster identification of risks.
Risk factors	Tool for identifying variable risk factors, raising awareness of risk, reducing risk, visualizing risk, incorporating relevant risk factors, transparency and relevance of factors, link between data and risk, and link between food and sugar.
Understanding of impacts	The impact of risk factors on disease vulnerability, visual representation of health status, understanding vulnerability, understanding necessary changes, understanding through visualization, simplicity and clarity of visualization, and understanding lifestyle changes.
Visualization and interpretation of results and the impact of technology use on lifestyle changes	
Personalized graphical display of results	Interactivity of visualization, real-time data visualization, visualization without explanation is not always informative enough, graphical representation of data, decision-making based on visualization, visual presentation as an advantage, color visualization, connection between colors and risk, display of changes, visual type, visualization as a communication tool, facilitating conversation.
Improved understanding of the interpretation of results	Easier understanding of data; simplification of complex data, visualization as a tool for clear presentation, advantage of images over numbers, awareness and impact on results, advantage of visualization over SCORE2^b^ tables, graphical representation of risk, visual displays enable faster and more accurate understanding without complicated procedures, transparency of visualization, ease of presentation for patients, and easier interpretation of results for patients.
Technology and usability	Significant impact of technology, usefulness of CGM^c^ for people with diabetes, self-monitoring and prevention, disease prevention, benefits for patients, and practicality.
Motivation and lifestyle changes	Patient motivation, changes in physical activity, motivation of the older adult population, motivation to change lifestyle, dietary changes, maintaining lifestyle changes, connection with meals, increased awareness, and control over food intake.
Identification of problems and challenges and recommendations for improvement	
Identification of problems or deviations	Identifying problems, realizing deviations, recognizing patterns, linking to results, reservations about use and lack of trust in technology, patients’ fear of using technology, negative interpretation of technology, and the challenge of affordability.
Opportunities for improvement and recommendations	Opportunities for improvement and emphasis on improvements, recommendation for wider implementation, link between decisions and health, potential for the future, application in other areas of health care, lack of education and knowledge and its inclusion, and improvement of visual tools.

a CVD: cardiovascular disease.

b SCORE2: Systematic Coronary Risk Evaluation 2.

c CGM: continuous glucose monitoring.

### Overview of the Findings

The 4 categories describe complementary aspects of participants’ experience rather than stages of a process. PCC describes the character of the consultation as participants experienced it: goal-oriented, individualized, and involving the patient in decisions. Knowledge and monitoring of risk factors describes what participants reported learning and noticing through Petal-X and CGM, including the recognition of deviations and the connection between everyday behavior and measurable indicators. Visualization and interpretation of results and the impact of technology use on lifestyle changes describes how the display itself was used and appraised, together with the dietary and physical activity changes that patients associated with it. Identification of problems and challenges and recommendations for improvement gathers the limitations that both groups reported, fear or mistrust of technology, the burden of continuous monitoring, variable digital literacy, information overload, and the cost of CGM, alongside their suggestions for addressing them.

Across all 4 categories, patients and nurses converged on the perceived usefulness of the visual display but differed consistently in emphasis. Patients spoke primarily about personal meaning, reassurance, and self-regulation; nurses spoke primarily about communication, the efficiency of the consultation, and the fit of the tool with existing workflows. Where the 2 groups diverged, this is made explicit in the sections that follow.

### Category 1: Person-Centered Care

#### Goal-Oriented Care and Individual Approach

Participants described consultations as more individualized and goal-focused than usual encounters, with more time for dialogue and tailoring recommendations to daily routines (eg, meal planning, physical activity, and family support). Nurses emphasized that visual displays supported more transparent communication and helped patients identify personally salient risk factors. Interpreted within the core category, these accounts suggest that the value of the consultation lay less in the visual tool itself than in how it reorganized the encounter around the individual: by making risk personally legible, the visualization created a shared reference point that shifted the interaction from information delivery toward negotiated, goal-oriented care. Patients and nurses agreed that a consultation supported by the visualization differed from a routine preventive encounter, but they located that difference in different places. For patients, the difference was one of attention and time: the consultation was experienced as being about them rather than about a presenting complaint. One participant contrasted it directly with her usual visits, noting that “the consultation was dedicated to me. We talked about me, my lifestyle and my habits. It took more time than my usual healthcare visits, where I normally go only because of specific problems,” and adding that she “felt more involved and received more information about my health” (P1; woman, aged 67 years, baseline SCORE2 risk 4%). Nurses rarely framed the change in terms of time; their emphasis fell on what the display did for the structure of the conversation, describing it as a way of making the assessment legible to the patient, in that “visualization is very useful in terms of transparency...easier to present to patients” (N2; ≥5 years of professional experience).

Read together, the 2 accounts suggest that the perceived value of the consultation lay less in the visual tool itself than in how it reorganized the encounter around the individual. By giving both parties a shared reference point, the visualization shifted the interaction away from the delivery of a risk figure and toward a negotiated discussion of daily routines, meal planning, and physical activity, which in several accounts extended to the involvement of family members. This is the sense in which participants described the care as goal-oriented: the goals were formulated during the consultation, and the display supplied the common ground on which they were agreed.

#### Involvement in Treatment

Both groups linked this reorganization of the consultation to a more active patient role, again with a difference of emphasis. Patients framed involvement in terms of partnership, control, and responsibility, describing a shift from recipient to interlocutor: one participant said that with this kind of care she “felt like a real conversation partner. We discussed what I could improve, and I had not experienced something like that before,” and connected seeing and understanding her data in real time with a “stronger sense of control over my health” (P3; woman, aged 58 years, baseline SCORE2 risk 7.8%). Nurses described the same shift from the outside, as a lowering of the threshold for participation, since with the visual display it was “easier for patients to get involved...easier to understand” (N3; ≥5 years of professional experience). Patients in turn linked this sense of control and responsibility to their willingness to follow the recommendations agreed during the consultation. It is worth noting that none of the reservations raised in the interviews concerned the shared discussion of results itself; where patients reported burden, it related to continuous self-monitoring, described under category 4, rather than to their involvement in decisions about their care.

### Category 2: Knowledge and Monitoring of Risk Factors for the Development of CVD

#### Monitoring Vital Functions and Detecting Deviations

Real-time monitoring (particularly CGM) was described as enabling rapid feedback and earlier recognition of deviations. Participants highlighted the importance of continuous access to data; nurses emphasized increased awareness and perceived patient independence. This pattern indicates that real-time monitoring did more than supply data; by compressing the interval between behavior and feedback, it appeared to support a sense of agency and self-regulation, which participants linked to sustained motivation rather than to a single moment of insight. Real-time monitoring, and CGM in particular, was the element of the intervention that patients raised most spontaneously. What they valued was less the data themselves than the continuous availability of feedback and the short interval between an action and its visible consequence. One participant described the sensor as a constant companion that also allowed retrospective reflection: “Those data were always on my side...when I saw that the glucose level was high, or when it increased, I was a little more careful to eat less...I could look back over the last 10 days and reflect on my state over time” (P1; woman, aged 67 years, baseline SCORE2 risk 4%). The color coding of the display was repeatedly described as doing interpretive work that numbers alone did not: “Numbers do not tell you enough, because you do not know exactly where the threatened area is. When it is visualized and turns orange or red, you experience it almost like a silent alarm, and then you approach lifestyle change more seriously” (P4; woman, aged 58 years, baseline SCORE2 risk 5.8%).

Nurses observed the same behavior from a professional standpoint but described it as a change in patients’ attentiveness and independence rather than as a personal experience, reporting that “patients monitor their condition more closely...more aware” (N2; ≥5 years of professional experience). Across both groups, therefore, monitoring was described as doing more than supplying data: by compressing the interval between behavior and feedback it appeared to support self-regulation, which participants linked to sustained attention to their habits rather than to a single moment of insight. The contrast with the annual preventive checkup, in which feedback arrives once and well after the behavior it reflects, was implicit in several patient accounts.

#### Identifying Risk Factors

Whereas CGM was associated with day-to-day feedback, Petal-X was associated with making the composition of long-term risk visible. Patients described the dashboard as the first occasion on which their risk profile had been presented in a form they could interpret; one participant recalled being able to “see for the first time how high my risk [was]...and what that means” (P3; woman, aged 58 years, baseline SCORE2 risk 7.8%). Nurses’ accounts converged with this, describing the display as making the contribution of individual modifiable factors transparent and relevant, and therefore easier to connect to a concrete recommendation within the consultation. What patients added to the nurses’ account was the personal meaning of that transparency: the value they described lay in relating the displayed factors to their own everyday behavior, rather than in the precision of the estimate itself.

#### Understanding Impacts

Participants described visualization as enhancing comprehension of how lifestyle factors influence risk and what changes are needed. Nurses similarly described visual feedback as supporting awareness and action. Taken together, these accounts frame comprehension as a mechanism rather than an outcome: translating abstract, probabilistic risk into a concrete visual form appeared to make the link between everyday choices and long-term risk actionable, consistent with the core category that connects visualization and digital tools to motivation and lifestyle change. Both groups described comprehension as the mechanism through which the visualization worked, rather than as an end point in itself. Patients contrasted the visual display with the numerical formats they had encountered previously: “Before, when I received results only as percentages, I could not fully imagine what those numbers actually meant for me. The visualization tool allowed me to see how my everyday decisions, such as diet and physical activity, directly affected my health. It was not just a number on paper, but a clear picture of how my choices influenced my risk” (P2; man, aged 63 years, baseline SCORE2 risk 10%). Nurses described the same effect in terms of its immediacy within the encounter, observing that with the visual display “patients immediately become aware” (N2; ≥5 years of professional experience). The difference between the 2 perspectives is largely one of timescale: nurses referred to comprehension achieved during the consultation, whereas patients described an understanding that persisted afterward and informed subsequent choices. Taken together, the accounts indicate that translating an abstract, probabilistic estimate into a concrete visual form was what made the link between everyday choices and long-term risk actionable for participants.

### Category 3: Visualization and Interpretation of Results and the Impact of Technology Use on Lifestyle Changes

#### Personalized Graphical Display and Interpretation

Interactivity was described as the feature that turned the display from a presentation into a discussion. Patients reported that reviewing the graphs together created an opening for asking questions and for proposing changes themselves: “When we reviewed the graphical displays of my results together, I could ask questions and suggest changes to my care plan. In the past I was often just a listener, but now I was truly involved in deciding about my health” (P2; man, aged 63 years, baseline SCORE2 risk 10%). Nurses valued the same interactivity for a different reason, namely, the speed with which it surfaced where change was possible; one nurse noted that the tool “very clearly illustrates deviations...opportunities for improvement” (N4; ≥5 years of professional experience). Nurses qualified this in an important respect: several observed that a visualization presented without accompanying explanation was not in itself sufficiently informative, and therefore the professional’s interpretation remained necessary. That qualification locates the benefit in the combination of display and dialogue rather than in the display alone, and it is consistent with the patients’ own emphasis on the conversation that the graphs made possible.

#### Technology and Usability

Appraisals of usability were broadly positive in both groups but were expressed in group-specific terms. Patients spoke of the technology as unproblematic in use, one stating simply that she was “very satisfied with the technology we used” (P3; woman, aged 58 years, baseline SCORE2 risk 7.8%). Nurses assessed usability against the tools they currently use, and it was in this comparison that the perceived advantage was most clearly articulated: “The table we use in the clinic is very comprehensive and time-consuming, because you have to look for each piece of data separately and compare it. With this tool, everything is together, explained and shown in one place, and it is visually simpler” (N3; ≥5 years of professional experience). The nurses’ positive appraisal was nevertheless conditional. They regarded the tool as practical and promising for preventive family medicine provided that it could be fitted into existing consultation workflows and that additional support was available for patients with lower digital literacy, conditions to which they returned when discussing implementation barriers.

#### Motivation and Lifestyle Changes

Patients’ accounts of behavior change were concrete and specific, and in several cases extended beyond the intervention period. One participant reported that he still followed certain guidelines and remained motivated, having “realised that my wellbeing has improved a lot and that I have much more energy than before” (P2; man, aged 63 years, baseline SCORE2 risk 10%). Another described a gradual rather than an abrupt change: “I changed the way I eat, especially sweets and fructose. I started to be very selective about what I eat. I also started going to the swimming pool and try to maintain walking as much as I can. The change was not sudden, but gradually I changed my diet a lot” (P4; woman, aged 58 years, baseline SCORE2 risk 5.8%). Nurses did not report outcomes of this kind, which they were not in a position to observe; their contribution was an account of why the tool might be motivating, namely, that it renders the room for improvement visible and concrete rather than abstract. Nurses were also more cautious than patients about how far this effect would reach, anticipating that motivation would be easier to elicit in younger, digitally confident patients than in older ones, a reservation examined in the next category.

### Category 4: Identification of Problems and Challenges and Recommendations for Improvement

#### Problems and Deviations

Implementation barriers included fear or mistrust of technology, the risk of negative interpretation, usability challenges for older users, affordability (especially for CGM), and the time burden of conventional tools. The barriers described by the 2 groups were largely distinct, reflecting their different positions in relation to the intervention. Patients described difficulties arising from continuous self-monitoring. These ranged from an initial apprehension about what the results would show, “I was a little scared of reality...maybe it is a little scary to compare it with reality, but on the other hand I would say it is more positive” (P1; woman, aged 67 years, baseline SCORE2 risk 4%), to the practical and attentional burden of wearing and following the sensor: “Constant monitoring was at times somewhat burdensome and stressful, because I was paying attention to the numbers the whole time...because my work is very active and I usually do not have my phone with me, it was a burden to always watch where my phone was so as not to lose the signal with the sensor” (P2; man, aged 63 years, baseline SCORE2 risk 10%). A related concern was the fear of overlooking something (P3; woman, aged 58 years, baseline SCORE2 risk 7.8%), and one participant described the monitoring becoming compulsive before settling: “it can quickly become an obsession. When I was wearing the glucose device, during the first few days I was constantly checking it. It was almost like a teenager who cannot get away from the phone. You have to find a balance” (P4; woman, aged 58 years, baseline SCORE2 risk 5.8%). Importantly, these accounts were not offered as reasons to reject the technology; the same participants also described the monitoring as motivating, and the difficulty they identified was one of calibration rather than of value.

The nurses’ concerns lay elsewhere, in feasibility and equity. They contrasted the tool favorably with the existing paper-based procedure, noting that with “SCORE2 tables, you need a lot of time and it is more likely that you make a mistake and then start over. Here it is much simpler and faster” (N5; ≥5 years of professional experience), but identified the digital divide as the principal limitation on who could benefit: “For the older population, it may be harder to motivate them to use this tool, whereas younger people, who are more digitally advanced, would probably understand more easily why it is useful” (N1; ≥5 years of professional experience). Nurses further raised the affordability and accessibility of CGM, which is not routinely reimbursed for people with no diabetes, and the risk that a patient might interpret a display negatively in the absence of professional support. Taken together, the patient-reported and nurse-reported barriers point in different directions for implementation: the former concerns how continuous monitoring is introduced and framed for the individual, whereas the latter concerns whether the approach can be delivered equitably within routine services.

#### Opportunities for Improvement

Suggested improvements focused on training and support, avoiding information overload, preserving the intuitive visual metaphor (flower), and extending use to other clinical areas. The suggestions made by each group followed from the barriers they had identified. Patients proposed extending the scope of what is measured and consolidating it in one place: “Maybe in the future it would be possible to measure something else, not only glucose. It would be useful if the same sensor or application could also include movement, steps and other aspects, so that everything would be visualized in one application” (P4; woman, aged 58 years, baseline SCORE2 risk 5.8%). The nurses’ recommendations were oriented toward wider adoption and the conditions for it, encompassing training and support for both staff and patients, the avoidance of information overload, and the preservation of the intuitive flower metaphor, which they regarded as central to the accessibility of the tool. Several also saw application beyond cardiovascular prevention: “I think this is a big bright spot for the future. It could also be used in other areas of health care, or in other fields, to motivate patients” (N2; ≥5 years of professional experience). Across the 2 groups, the recommendations were complementary: patients focused on the completeness and integration of the information they receive, and nurses focused on the organizational preconditions for using that information in routine practice.

Figure 1 presents a conceptual map of the categories and subcategories identified in the analysis, together with the overarching theme that runs across them. The map summarizes the descriptive structure of the findings; the layout does not imply a causal, temporal, or hierarchical sequence between the categories.

**Figure 1.**
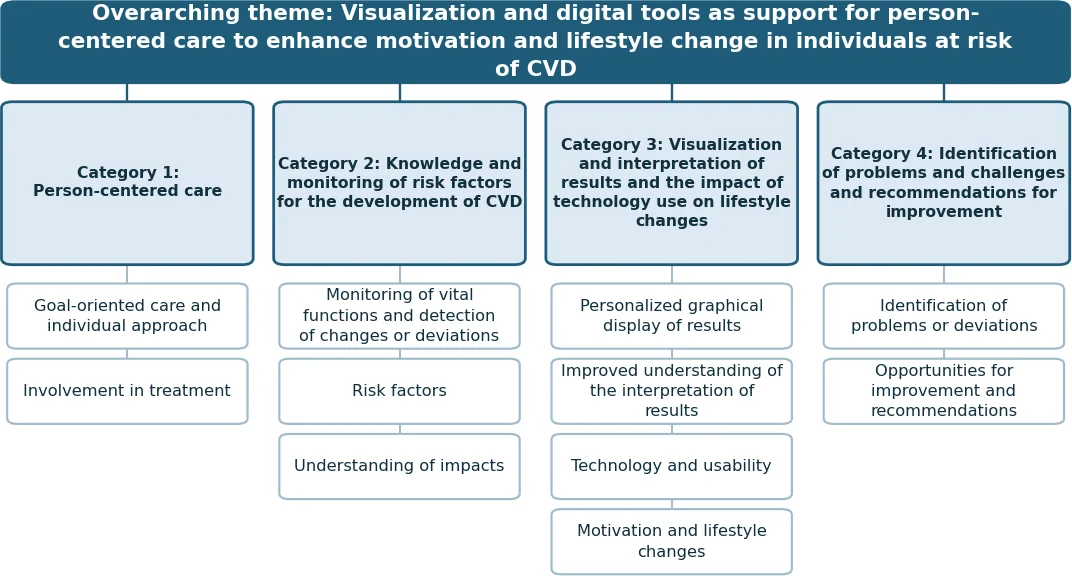
Conceptual map of the categories and subcategories describing how patients and nurses experienced the Petal-X visualization and continuous glucose monitoring. Categories and subcategories are shown as a descriptive structure; the layout does not imply a causal, temporal, or hierarchical sequence between categories. CVD: cardiovascular disease.

### Relation to the Parallel Quantitative Study

This paper reports the qualitative phase of a sequential mixed methods study; the quantitative randomized controlled trial is reported separately. We did not statistically integrate the 2 datasets in this paper. Instead, consistent with sequential mixed methods designs, the qualitative findings are connected to the trial at the level of interpretation: they help explain the mechanisms that may underlie the trial results, namely, greater understanding, involvement, self-perceived control, and motivation supported by visualization and real-time feedback. The literature review informed the selection of technologies and risk factors incorporated into the intervention.

## Discussion

### Principal Findings

In this qualitative study, both nurses and patients experienced interactive CVD risk visualization (Petal-X), particularly when combined with CGM, as a meaningful support for PCC. Across 4 categories and 11 subcategories, participants described a clearer understanding of risk factors, more individualized and shared consultations, a greater sense of involvement and control, and stronger motivation for dietary and physical activity change, which some patients reported maintaining after the study. At the same time, participants and nurses highlighted important barriers, including variable digital literacy among older users, fear or mistrust of the technology, the burden of continuous self-monitoring, potential information overload, workflow and training needs, and the affordability and accessibility of CGM. Qualitative analysis of interviews with nurses and participants reveals key aspects of PCC that were identified during the introduction of new technologies in the family medicine practice. The participants expressed satisfaction with the individualized approach, which includes adapting care to the needs and wishes of individuals and actively involving patients in the treatment process.

The perceptions of nurses and participants regarding the use of cardiovascular risk visualization (Petal-X) and CGM are crucial for understanding how effectively these technologies improve management and motivation for lifestyle changes. The categories that enable us to answer the research question are the following: goal-oriented care and an individual approach, involvement in treatment, monitoring of vital functions and technology, motivation and lifestyle changes, understanding of impacts and risk factors, and opportunities for improvement. The category that most strongly organized participants’ accounts was PCC.

PCC, supported by technologies that enable personalization and visualization of health data, emerged in our study as an effective strategy for encouraging lifestyle changes among patients in the family medicine setting. Such an approach not only enhances the quality of care but also fosters collaboration between patients and health care professionals, ultimately contributing to improved long-term health outcomes. These findings are consistent with previous interventions that have focused on patient education, structured follow-up and enhanced support for self-management, such as programs for disease self-management education [28,29], structured discharge plans with tailored follow-up [30], and lifestyle counseling, home visits, and telephone support led by health care professionals [31].

Our results suggest that interventions that combine education, personalized care plans, and lifestyle adaptation to the individual’s cardiovascular risk could be further strengthened by incorporating visual risk displays [9,32-35], mobile apps [36-39], and additional biometric measurements. CVDs remain a leading cause of morbidity and mortality worldwide, and early identification and engagement of at-risk individuals are crucial for effective prevention. In this context, digital tools that translate complex prognostic information into intuitive, patient-friendly formats align strongly with the principles of PCC and offer a promising route to more effective preventive strategies.

A central finding of our qualitative analysis is that goal-oriented care and an individualized, data-driven approach are key to tailoring care for patients at risk of CVD. Participants consistently reported that they experienced the consultation as more holistic, with more time for conversation and shared decision-making about their health. This tailored approach took into account specific needs, values, and goals, and included adjustments to diet, physical activity, and daily routines, often with the support of family members. New forms of visualization, such as interactive graphs and personalized analyses of risk factors, were perceived as particularly helpful. They improved communication, enhanced understanding of patients’ health status, and encouraged them to participate actively in planning their care.

The Petal-X visualization dashboard, developed and used in our study, represents a concrete step toward augmenting SCORE2 with an explanation layer and operationalizing PCC in routine practice. By providing a clear, structured overview of individual CVD risk factors, Petal-X made the risk profile more transparent and easier to interpret for both patients and nurses. Visualizations increased the sense of overview and control among participants, allowing them to see how different risk factors contribute to their overall risk and what might change if they modify certain behaviors [9,27,32,34,35]. Nurses emphasized that this approach improved the quality of consultations, simplified workflows, and made it easier to involve patients in setting realistic, personalized goals, findings that echo positive outcomes reported in other technology-supported interventions [40-43].

In addition to visualization, the use of CGM, typically reserved for people with diabetes, introduced a dynamic and highly engaging element to lifestyle counseling. Participants carried the CGM device with them and were able to observe real-time changes in their glucose levels in response to food intake, physical activity, stress, and other daily situations. This immediate feedback created a direct link between behavior and physiological response, which several participants identified as a strong motivator for changing and maintaining healthier habits. Similar benefits of real-time monitoring and digital feedback loops have been documented in studies using mobile apps and wearable technologies to support self-management and lifestyle modification [28,29,36-39,41].

Overall, our qualitative findings indicate improvements in patients’ understanding of their health status, awareness of CVD risk, satisfaction with the integration of technologies, and willingness to participate in planning their lifestyle changes. Participants reported an increased motivation to continue monitoring and adjusting their lifestyle even after the study ended. From the perspective of nurses, the combined use of Petal-X and CGM supported more structured, person-centered consultations and facilitated the integration of preventive interventions into everyday practice. These experiences are consistent with evidence from other technology-enhanced interventions, which have demonstrated positive outcomes when new tools are embedded in supportive, PCC models [42-44].

The Slovenian context adds an important system-level dimension to these findings. Currently, there is no standardized digital tool that integrates multiple solutions for both nurses and patients, nor is there a unified national electronic health record system that would seamlessly support such approaches. Our implementation of Petal-X and CGM within this fragmented digital environment underscores the potential of these tools to enhance care even in the absence of a comprehensive national infrastructure. At the same time, it underscores the necessity for national guidelines on integrating new technologies into health care, particularly in the areas of prevention and chronic disease management.

Finally, lifestyle modification remains one of the most important modifiable risk factors for reducing the prevalence of CVD risk, making research and innovation in this area especially valuable in Slovenia. Our results suggest that lifestyle change is closely linked to patients’ perception of PCC, especially the experience of an individualized approach, personalized visualization of risk, and a holistic, respectful consultation process. The inclusion of new technologies in lifestyle interventions can increase patient motivation, enable real-time monitoring of health status, and support higher-quality PCC. Further research should explore how such tools can be scaled up, integrated into national digital ecosystems, and combined with established interventions, such as education, structured follow-up, and community-based support [28,30,31], to strengthen prevention and long-term management of CVD risk.

### Future Research

Several directions for future research follow from these findings. First, larger studies across multiple primary care settings are needed to test whether the perceived benefits of risk visualization and CGM extend to more diverse populations and to evaluate how the tools can be scaled and embedded in routine workflows and in national digital health infrastructure, including electronic health record systems. Second, because CGM carries nontrivial costs, future work should include an affordability and risk-benefit analysis from a public health perspective, so that decisions about wider adoption weigh the added value of continuous monitoring against its cost and its equity implications for patients who cannot afford such devices. Third, given the focus on secondary prevention and PCC, future studies should more explicitly incorporate patients’ own preferences, needs, and values, for example, through a structured “What Matters to You?” approach, so that digitally supported risk communication is aligned with what patients themselves consider important. Finally, longer follow-up is needed to determine whether the motivation and behavioral changes that participants described translate into the long-term sustainability of lifestyle change and into measurable cardiovascular risk reduction over time.

### Limitations

Several limitations should be considered when interpreting these findings. First, this was a qualitative descriptive study based on semistructured interviews with a small sample of nurses (n=6) and intervention participants (n=6) recruited by total population sampling from a closed, predefined trial cohort, which supports depth of description but limits transferability to broader primary care populations and settings. Second, although recruitment was open across Slovenia, the promotional campaign and implementation were concentrated in a single Community Health Centre in central Slovenia, so local organizational routines and context may have shaped participants’ experiences and perceptions. In addition, recruitment from a single center, combined with the small sample size, resulted in a relatively homogeneous sample, which may further limit the transferability of the findings. Third, the study relied on self-reported experiences, which may be influenced by recall bias and social desirability. Finally, perceptions of Petal-X and CGM may reflect novelty effects and practical implementation constraints; importantly, participants and nurses also reported barriers such as variable digital literacy (particularly among older users), fear or mistrust of technology, potential information overload, workflow integration needs, and affordability or access limitations of CGM, which may affect scalability and equity of implementation.

A further limitation, and one that follows directly from the sampling strategy described, concerns who was eligible to be interviewed. Both samples were restricted to individuals who had completed their part of the intervention: patients who had completed the intervention arm, as defined in the Methods section, and nurses who had completed the familiarization with the intervention and remained in the preventive service throughout the trial period. Patients who withdrew, who discontinued sensor wear, or who did not attend the final assessment were by definition unavailable for interview, and their reasons for discontinuing, which may include precisely the burdens, anxieties, and usability problems that an intervention of this kind is most likely to generate, are therefore not represented in our data. Completer-only sampling plausibly selects for higher engagement with digital tools, greater tolerance of continuous self-monitoring and of the practical and attentional demands of wearing a sensor, and a degree of comfort with self-quantification that may not be typical of the wider primary prevention population. The barriers reported here, including the initial apprehension about what the results would show, the compulsive checking described by one participant, and the burden of keeping a smartphone within range of the sensor, should therefore be read as difficulties reported by people who nevertheless persisted to the end of the intervention, and they are likely to understate both the frequency and the intensity of the problems that would arise in an unselected population.

A related but distinct consideration applies to the nurses. As described in the Methods section, all trial consultations were delivered by the first author (AS), and the nurses interviewed had been familiarized with the intervention and the Petal-X dashboard rather than having used them with their own caseload in routine practice. Their appraisals of usability, workflow fit, and feasibility are therefore the anticipatory judgments of informed prospective users rather than reports of sustained delivery, and they may not capture the frictions that would emerge once such a tool is embedded in everyday consultations, nor the adaptations that practitioners make over time. Equally, the nurses who agreed to be interviewed were those who had engaged with the familiarization offered at the center, which may again select for interest in digital tools. Research in which nurses themselves deliver visualization-supported consultations over a sustained period is needed before conclusions about routine implementation can be drawn. It should also be noted that the interviewed patients were somewhat older than the intervention arm as a whole (mean 57.8 vs 51.9 years), so that the older group whose digital literacy the nurses identified as a barrier was, if anything, overrepresented among the interviewees, although these were older adults who had nonetheless completed the intervention successfully. Studies that deliberately sample noncompleters, partial users, and individuals who decline such interventions are needed to characterize the full range of responses to visualization-supported and CGM-supported cardiovascular prevention.

### Conclusions

This qualitative study demonstrates that interactive CVD visualization (Petal-X), particularly when combined with real-time glucose feedback via CGM, can enhance key elements of PCC in primary prevention. Both patients and nurses described that those personalized visual displays improved comprehension of risk factors, supported shared decision-making, and increased patients’ sense of involvement and control, mechanisms that were perceived as essential drivers of motivation and lifestyle change.

At the same time, implementation challenges were identified, including variable digital literacy (particularly among older users), concerns about information overload, the need for training of health care professionals, and affordability and accessibility of devices such as CGM. Future work should evaluate how these tools can be integrated into routine workflows and national digital infrastructure, and how they can be scaled in a way that preserves usability, equity, and sustained lifestyle benefits.

## Supplementary material

10.2196/92101 Multimedia Appendix 1 Interview guide for registered nurses.

10.2196/92101 Multimedia Appendix 2 Interview guide for patients.

10.2196/92101 Checklist 1 COREQ checklist.

## Abbreviations

CGM: continuous glucose monitoring
COREQ: Consolidated Criteria for Reporting Qualitative Research
CVD: cardiovascular disease
PCC: person-centered care
RN: registered nurse
SCORE2: Systematic Coronary Risk Evaluation 2
