# Supplementary material for: Risk Visualization With Petal-X and Continuous Glucose Monitoring to Support Person-Centered Care and Lifestyle Change in Cardiovascular Prevention: Qualitative Descriptive Study
Source: JMIR Hum Factors. 2026 Oct 13;13:e92101. doi: 10.2196/92101 (PMC13660584; doi:10.2196/92101)
Supplement: Supplementary file 1 [file humanfactors-v13-e92101-s001.docx]

# **Multimedia Appendix 1. Interview guide for registered nurses**

Semi-structured interview guide used with registered nurses who had used the Petal-X cardiovascular disease (CVD) risk visualization dashboard and continuous glucose monitoring (CGM) during the study. The guide was developed from the literature review and the findings of the quantitative phase; follow-up and probing questions were used as needed.

The location and time of the interview are arranged individually with each participant. Each participant is individually informed about the purpose and course of the study, as well as about the possibility of withdrawing from the interview.

Before the start of the interview, the participant provides oral and written consent to participate in the study.

Each participant is assigned a code to ensure anonymity and confidentiality of the data in the study.

**1. Interview code:** ___________

**2. Basic participant information:**

a) **Gender:**

- Female
- Male
- Non-binary

b) **Age (years):** ___________

**3. Topics for discussion**

Could you please tell me how you envision the care process and the presentation of results to patients using cardiovascular disease risk visualization?

Could you please tell me how, in your opinion, the visualization used during the study compares with SCORE2 tables, particularly regarding ease of use and understandability for healthcare professionals and patients?

Could you please tell me how you understand the visual tool? Do you think these tools could be used to show patients improvement or motivate them to change their lifestyle?

Could you please tell me what impact you think these tools have on improving care and encouraging lifestyle changes among patients? Could you also explain this with an example?

Could you please tell me whether you think the use of these tools would contribute to higher-quality care and greater involvement of the patient in the care plan?

Could you please describe the challenges of using these technologies in your practice? What advantages or disadvantages does the use of these technologies bring for you or for patients?

Could you please tell me whether there are any functionalities or information that you would add to or change in the visualization tool? How, in your opinion, could the user experience with this tool be improved?

Could you please tell me whether there is anything else you would like to add regarding the use of these tools and care in family medicine practices?

Thank you for your participation.
