# Supplementary material for: Risk Visualization With Petal-X and Continuous Glucose Monitoring to Support Person-Centered Care and Lifestyle Change in Cardiovascular Prevention: Qualitative Descriptive Study
Source: JMIR Hum Factors. 2026 Oct 13;13:e92101. doi: 10.2196/92101 (PMC13660584; doi:10.2196/92101)
Supplement: Supplementary file 2 [file humanfactors-v13-e92101-s002.docx]

# **Multimedia Appendix 2. Interview guide for patients**

Semi-structured interview guide used with patients who had taken part in the intervention arm combining the Petal-X CVD risk visualization dashboard with real-time (continuous) glucose monitoring. The guide was developed from the literature review and the findings of the quantitative phase; follow-up and probing questions were used as needed.

The location and time of the interview are arranged individually with each participant. Each participant is individually informed about the purpose and course of the study, as well as about the possibility of withdrawing from the interview.

Before the start of the interview, the participant provides oral and written consent to participate in the study.

Each participant is assigned a code to ensure anonymity and confidentiality of the data in the study.

**1. Interview code:** ___________

**2. Basic participant information:**

a) **Gender:**

- Female
- Male
- Non-binary

b) **Age (years):** ___________

**3. General topics for discussion**

Could you please tell me how you would describe the care and the presentation of results using cardiovascular disease risk visualization? How did these tools influence your understanding of your health condition?

Could you please tell me how you understand the visual tool that was used to present your cardiovascular disease risk? Could you please explain this with an example?

Could you please tell me how you experienced the care? Did you perhaps feel more involved compared with previous care experiences? Could you please explain this with an example?

As a user of modern technology during the intervention, do you think that the visualization tool and the possibility of real-time monitoring of blood glucose levels contributed to improved person-centred care and lifestyle change? Could you please explain this with an example, particularly if it influenced any lifestyle changes?

Could you please describe the positive and negative aspects of the visualization and the possibility of real-time monitoring of blood glucose levels that you may have noticed?

Could you please tell me whether you feel that your thinking about lifestyle change has changed in any way? Could you explain this with an example?

Could you please tell me whether there is anything else you would like to add regarding the technologies used during your care and your involvement in the process and individual work?

Thank you for your participation.
