# Supplementary material for: Risk Visualization With Petal-X and Continuous Glucose Monitoring to Support Person-Centered Care and Lifestyle Change in Cardiovascular Prevention: Qualitative Descriptive Study
Source: JMIR Hum Factors. 2026 Oct 13;13:e92101. doi: 10.2196/92101 (PMC13660584; doi:10.2196/92101)
Supplement: Supplementary file 3 [file humanfactors-v13-e92101-s003.docx]

# **COREQ checklist**

Consolidated Criteria for Reporting Qualitative Research (COREQ). Based on Tong A, Sainsbury P, Craig J. Int J Qual Health Care. 2007;19(6):349-357.

| **No.** | **Item** | **Guide Questions/Description** | **Reported on Page #** |  |  |
| --- | --- | --- | --- | --- | --- |
| **Domain 1: Research team and reflexivity** | | | |  |  |
| 1 | Interviewer/facilitator | Which author/s conducted the interview or focus group? | Page 22 (Authors’ Contributions: “AS collected the data”) |  |  |
| 2 | Credentials | What were the researcher’s credentials? E.g., PhD, MD | Page 5 (registered nurse, MSc) |  |  |
| 3 | Occupation | What was their occupation at the time of the study? | Page 5 (doctoral researcher and teaching assistant, University of Maribor) |  |  |
| 4 | Gender | Was the researcher male or female? | Page 5 (female) |  |  |
| 5 | Experience and training | What experience or training did the researcher have? | Page 5 (trained in qualitative research methods) |  |  |
| Relationship with participants | | | |  |  |
| 6 | Relationship established | Was a relationship established prior to study commencement? | Page 5 (no prior clinical or supervisory relationship) |  |  |
| 7 | Participants' knowledge of the interviewer | What did the participants know about the researcher? e.g. personal goals, reasons for doing the research? | Page 5 (informed of background and doctoral research project) |  |  |
| 8 | Interviewer characteristics | What characteristics were reported about the interviewer/facilitator? e.g. Bias, assumptions, reasons and interests in the research topic | Page 5 (assumptions, reflexive memos, peer debriefing); also Page 6 |  |  |
| **Domain 2: Study design**  Theoretical framework | | | |  |  |
| 9 | Methodological orientation and theory | What methodological orientation was stated to underpin the study? e.g. grounded theory, discourse analysis, ethnography, phenomenology, content analysis | Pages 1 and 4 (grounded theory; Corbin & Strauss) |  |  |
| Participant selection | | | |  |  |
| 10 | Sampling approach | How were participants selected? e.g., purposive, convenience, consecutive, snowball | Pages 4–5 (purposive – theoretical sampling) |  |  |
| 11 | Method of approach | How were participants approached? e.g., face-to-face, telephone, mail, email | Page 4 (recruitment / promotional campaign); specific mode of approach (email) By email (not stated in the manuscript text) |  |  |
| 12 | Sample size | How many participants were in the study? | Pages 4–5 and 7 (6 nurses, 6 patients) |  |  |
| 13 | Non-participation | How many people refused to participate or dropped out? Reasons? | N/A |  |  |
| Setting | | | |  | Where was the data collected? e.g., home, clinic, workplace |
| 14 | Setting of data collection | Where was the data collected? e.g., home, clinic, workplace | Page 4 |  |  |
| 15 | Presence of non-participants | Was anyone else present besides the participants and researchers? | N/A (No) |  |  |
| 16 | Description of the sample | What are the important characteristics of the sample? e.g. demographic data, date | Page 7 |  |  |
| Data collection | | | |  |  |
| 17 | Interview guide | Were questions, prompts, and guides provided by the authors? Was it pilot tested? | Page 5; guides in Multimedia Appendix 1 & 2 (Page 23). Pilot testing not reported |  |  |
| 18 | Repeat interviews | Were repeat interviews carried out? If yes, how many? | N/A (No) |  |  |
| 19 | Audio/visual recording | Did the research use audio or visual recording to collect the data? | Page 6 (audio-recorded) |  |  |
| 20 | Field notes | Were field notes made during and/or after the interview or focus group? | N/A |  |  |
| 21 | Duration | What was the duration of the interviews or focus group? | N/A |  |  |
| 22 | Data saturation | Was data saturation discussed? | Pages 4–6 (theoretical saturation) |  |  |
| 23 | Transcripts returned | Were transcripts returned to participants for comment and/or correction? | Page 6 (yes – member checking) |  |  |
| **Domain 3: Analysis and findings**  **Data analysis** | | | |  |  |
| 24 | Number of data coders | How many data coders coded the data? | Page 22 (Authors’ Contributions: LG, GS, AS performed the analysis) |  |  |
| 25 | Description of the coding tree | Did the authors provide a description of the coding tree? | Page 6 (open/axial/selective coding) and Table 1 (Pages 8–10) |  |  |
| 26 | Derivation of themes | Were themes identified in advance or derived from the data? | Page 6 (derived from the data) |  |  |
| 27 | Software | What software, if applicable, was used to manage the data? | N/A |  |  |
| 28 | Participant checking | Did participants provide feedback on the findings? | Page 6 (member checking of transcripts); feedback on findings not separately reported |  |  |
| Reporting | | | |  |  |
| 29 | Quotations presented | Were participant quotations presented to illustrate the themes/findings? Was each quotation identified? e.g., participant number | Pages 11–14; identifiers (P, N) explained on Page 6 |  |  |
| 30 | Data and findings consistent | Was there consistency between the data presented and the findings? | Pages 7–15 (Results) |  |  |
| 31 | Clarity of major themes | Were major themes clearly presented in the findings? | Pages 7–14 (core category and four categories; Table 1) |  |  |
| 32 | Clarity of minor themes | Is there a description of diverse cases or a discussion of minor themes? | Pages 13–14 (negative/diverse cases) and Table 1 (subcategories) |  |  |
